# A Novel Konjac Powder with High Compressibility, High Water-Holding Capacity, and High Expansion Force

**DOI:** 10.3390/foods14020211

**Published:** 2025-01-11

**Authors:** Qianru Li, Jiabin Qin, Hongshan Liang, Jing Li, Shuxin Ye, Mahmoud Youssef, Yuanyuan Chen, Bin Li

**Affiliations:** 1 College of Food Science and Technology, Huazhong Agricultural University, Wuhan 430070, China; lihah10@foxmail.com (Q.L.); jiabinhf@163.com (J.Q.); lianghongshan@mail.hzau.edu.cn (H.L.); lijingfood@mail.hzau.edu.cn (J.L.); m13628691513@163.com (S.Y.); mahmoudyoussef@azhar.edu.eg (M.Y.); yychen@webmail.hzau.edu.cn (Y.C.); 2 Shenzhen Institute of Nutrition and Health, Huazhong Agricultural University, Shenzhen 518000, China; 3 Shenzhen Branch, Guangdong Laboratory for Lingnan Modern Agriculture, Genome Analysis Laboratory of the Ministry of Agriculture, Agricultural Genomics Institute at Shenzhen, Chinese Academy of Agricultural Sciences, Shenzhen 518000, China; 4 Food Science and Technology Department, Faculty of Agriculture, Al-Azhar University, Cairo 11651, Egypt

**Keywords:** konjac powders, moisture treatment, single and combined drying methods, moisture transition points, hydration properties, compressibility

## Abstract

The inherent physico-chemical properties of commercial konjac powders often limited their application across various industries. While existing modification techniques had produced konjac powders with diverse physical attributes, these methods were frequently associated with high costs and environmental concerns. Hence, there was a critical need to develop a cost-effective, environmentally friendly, and straightforward method for modifying konjac powders. This study investigated the effects of limited moisture modification combined with drying methods on the key physical properties of konjac powders using a comprehensive set of analytical techniques. The results demonstrated that the processed konjac powders exhibited enhanced hydration properties and compressibility. Notably, moisture modification at 54.04%, combined with vacuum freeze-drying (VFD), resulted in konjac powders with a loose, porous microstructure (porosity: 75.54%) and good tablet-forming properties, significantly exceeding that of the control group. Additionally, the combination of vacuum drying (VD, 17 h) and VFD (2 h) significantly improved the water-holding capacity (154.54 g/g) and expansion force (109.97 mL/g) of the konjac powders. This study provided a sustainable, safe, economical, and easily scalable method for tailoring the physical properties of konjac powders. The modified konjac powders developed here were suitable for applications requiring high hydration properties or direct powder compression.

## 1. Introduction

The global shift in dietary habits, the decline in fiber intake, has led to a significant rise in the prevalence of chronic diseases, particularly cardiovascular disease. This trend underscores the critical role of dietary fiber in maintaining overall health [1,2]. Dietary fiber is a well-established essential nutrient that offers many health benefits, including anti-obesity effects, glycemic control, and cholesterol reduction [3,4]. Clinical studies have demonstrated that high-fiber diets are associated with substantially reducing mortality risk and the incidence of chronic illnesses [5,6]. Konjac glucomannan (KGM), a primary component of commercial konjac powders, accounts for more than 90% of their composition. As a soluble dietary fiber, KGM exhibits excellent physico-chemical properties and notable biological activities, making it widely applicable in the food and packaging industries.

Despite the distinct physico-chemical properties of konjac powders, native konjac powders exhibited a limited range of functionalities, which might not fulfill the requirements of various industrial applications. For instance, the high viscosity and swelling rate of native konjac powders can pose challenges in specific contexts. Additionally, their high plasticity and poor compressibility often resulted in low-quality tablets, limiting their usability in certain formulations [7]. Consequently, konjac powders were commonly modified to broaden their functional versatility for diverse applications. To address these limitations and enhance the applicability of konjac powders, researchers had developed various modification techniques to tailor their physical properties to meet specific industrial demands. Traditional modification approaches predominantly involved physical, chemical, or enzymatic methods [8,9,10]. For example, Yin et al. (2020) employed endo-1,4-β-mannanase treatment to produce konjac powders with reduced viscosity and enhanced prebiotic activity, improving their physico-chemical properties and expanding their suitability for practical applications [11]. However, these conventional methods had inherent drawbacks. First, physical and enzymatic modifications often required substantial energy inputs, raising concerns about their economic viability and environmental sustainability. Second, chemical modifications, while effective, might introduce novel molecules that could pose safety concerns, particularly in food-related applications [12,13]. Third, most existing modification methods necessitate dissolving konjac powders in water, as these processes typically occur in the sol state [14,15]. Given that the primary component of konjac powders, konjac glucomannan (KGM), was the most viscous plant polysaccharide known, its high viscosity led to a low concentration of konjac powder in the sol state, thereby reducing process efficiency and increasing costs [16]. In light of these challenges, developing novel modification strategies for konjac powders has become an urgent priority. An ideal solution should maintain the macrostructural conditions of konjac powders, using environmentally friendly, safe, economical, and simple equipment to adjust its physical properties and expand its potential applications in various industries.

The drying process is a simple and versatile technique that can be easily implemented using existing equipment, making it a fundamental production step in industrial applications. During the drying process, water is removed from the sample through evaporation or sublimation, leading to complex changes in water-binding sites and the surface structure of konjac powders. These changes significantly influence the physico-chemical properties of the material. Currently, drying treatments are widely employed for the physical modification of various dietary fibers. For instance, vacuum freeze-drying (VFD) has been shown to enhance the hydration properties, adsorption capacity, prebiotic activity, and other functional characteristics of dietary fibers [17,18]. Similarly, vacuum drying has been reported to reduce starch’s swelling power and solubility [19] and increase coffee fiber’s microstructural porosity and bioactivity [20]. These findings demonstrate that drying treatments are a practical approach for modifying the physico-chemical properties of dietary fibers. Despite the proven efficacy of drying methods in modifying other dietary fibers, limited research has been conducted on their application for modulating the physical properties of konjac powders.

A review of the available literature revealed limited research on using the limited moisture modification combined with drying methods to modify the physical properties of konjac powders without compromising their macrostructure. To our knowledge, only one patent had reported on applying the limited moisture modification combined with hot air drying to improve konjac powders’ swelling, brewing properties, and palatability. The results indicated that this treatment extended the swelling time of konjac particles, improved brewing properties, and enhanced palatability, effectively addressing challenges such as short swelling times, clumping, and poor taste in konjac powders. However, this patent did not investigate the effects of the treatment on other critical characteristics of konjac powders, nor did it systematically explore the influence of different drying methods on their physical properties. Addressing this research gap, the present study was the first to systematically evaluate the effects of the limited moisture modification (moisture contents of konjac powders that retained their macrostructure after treatment) combined with various drying methods on the microstructure and key physical properties of konjac powders. These properties included hydration properties, dissolution characteristics, and powder compressibility. This research aims to develop a simple, economical, and environmentally friendly physical modification method that preserves the macrostructure of konjac powders while tailoring their properties to meet the requirements of diverse industrial applications and production needs.

## 2. Materials and Methods

### 2.1. Materials

Konjac powders (purity > 90%, molecular weight 4.475 × 10^5^ Da) were purchased from Hubei Qiang sen Konjac Co., Ltd. (Wuhan, China). Microcrystalline cellulose and fructooligosaccharide were obtained from Jie heng Biotechnology Co., Ltd. (Zhengzhou, China).

### 2.2. Preparation of Konjac Powders with Different Ranges of Moisture Contents

Native konjac powders and distilled water were mixed in specific ratios of 1:0.4, 1:0.6, 1:0.8, and 1:1.2 (powders/water) to prepare konjac powders with varying initial moisture contents. Briefly, a precise mass of konjac powders was weighed using a mortar, followed by adding a predetermined mass of distilled water according to the specified ratio. The mixture was vigorously stirred until achieving a homogeneous consistency, and then it was allowed to stand for 5 min. After mixing, a defined mass of the prepared konjac powders was weighed, and the moisture contents were determined using an HE 53/02 Rapid Moisture Analyzer (METTLER TOLEDO, Shanghai, China). The measured moisture contents were 29.32%, 37.80%, 44.79%, and 54.04%, corresponding to the aforementioned powders-to-water ratios [21].

### 2.3. Modification of Konjac Powders Using Different Drying Methods

#### 2.3.1. Modification of Konjac Powders by Vacuum Freeze-Drying, Hot Air Drying, and Vacuum Drying Methods

Konjac powders with a certain moisture content, as prepared in Section 2.2, were placed in a blast drying oven (50 °C, 5 h) (Wuhan Delixiang Instrument Co., Ltd., Wuhan, China), a vacuum drying oven (50 °C, 20 h) (Tianjin Tester Instrument Co., Ltd., Tianjin, China), and a vacuum freeze-dryer (−40 °C, 24 h) (Beijing Songyuan Huaxing Technology Development Co., Ltd., Beijing, China), respectively, until the moisture content of the konjac powders reached 4–5%.

#### 2.3.2. Modification of Konjac Powders Using Different Combined Drying Methods

Konjac powders with a certain moisture content were prepared in Section 2.2 and dried in stages using the combined drying methods until the final konjac powders had a 4–5% moisture content. The specific drying parameters are shown in Table 1.

#### 2.3.3. Modification of Konjac Powder Using Different Moisture Transition Points

Konjac powders with predetermined moisture contents, prepared as described in Section 2.2, were subjected to stage-drying using a combination of vacuum drying (VD) and vacuum freeze-drying (VFD). The drying process was designed to control the duration of the VD and VFD phases, with six combinations applied: 17–2 h, 14–5 h, 11–8 h, 8–11 h, 5–14 h, and 2–17 h (VD-VFD, respectively). The objective was to produce konjac powders with varying moisture transition points while ensuring a similar final moisture content of approximately 6–7% in the end product.

### 2.4. Particle Size Distribution Measurement

Due to the special characteristics of the samples, which could not be determined by laser particle size analyzer, this experiment referred to the sieving method described by Khullar et al., with slight modification for the determination of particle size distribution [22,23].

A certain amount of konjac powders was weighed and recorded as M_2_ and then placed on a series of sieves (40, 60, 80, 100, 160, and 200 mesh). The sieves vibrated until no powder came out from underneath the sieve. Finally, the interstitial material of each sieve was taken, weighed, and recorded as M_1_, repeated three times. The percentage of particle size of each sieve was calculated using the equation below:

(1) $$Percentage\;of\;particle\;size\;(\%)=M_{1}/M_{2}\times 100$$

### 2.5. Physico-Chemical Property Determination

#### 2.5.1. Expansion Force

According to da Silva et al. (2020), the expansion force of konjac powders was determined with minor modifications [24].

Briefly, 0.05 g of konjac powders (denoted as M_1_) were placed in a 10 mL measuring cylinder. Then, 9.95 g of distilled water was added. After that, the mixture was mixed well using a magnetic stirrer. Finally, the mixture was left to stand for 24 h at room temperature. The volume of expanded konjac powders was recorded and denoted as V_2_, repeated thrice. The expansion force was calculated using the equation below:

(2) $$Expansion\;force\;(mL/g)=V_{2}/M_{1}$$

#### 2.5.2. Water-Holding Capacity

The water-holding capacity of konjac powders was determined using the method described by Yan et al. (2019) with minor modifications [25].

Accurately, 19.9 g of distilled water was added to a 50 mL centrifuge tube, a magnet was placed, and 0.1 g of konjac powders were added while stirring for 6 h. Then, the tube sample was placed in a high-speed centrifuge (Xiangyi, Hunan, China) at 10,000 r/min for 20 min. The supernatant was carefully poured off and then weighed after being semi-inverted for 30 min, and the wet weight of the precipitate, M_1_, was recorded. Subsequently, the precipitate was transferred to the blower oven at 55 °C to dry for 24 h (to reach a constant weight), and the precipitate was weighed. The dry weight of the precipitate was recorded as M_2_ and repeated three times. Water-holding capacity was calculated using the equation below:

(3) $$Water-holding\;capacity\;(g/g)\;=\;(M_{1}\;-\;M_{2})/M_{2}$$

#### 2.5.3. Swelling Ratio

The swelling ratio (SR) of konjac powders was examined according to the method by Li et al. (2021) and Luo et al. (2019), with slight modifications [26,27].

Accurately, 1.00 g of treated/untreated konjac powders were placed into a uniform-sized double nylon cloth tea bag, then weighed and recorded as M_1_. The tea bags were placed in a beaker containing 50 g of distilled water, taken out according to a certain sampling time, and weighed by wiping off the excess water from the surface (a single time point was recorded as M_2_) for a total of 2 h. The tea bags were then sealed by sealing the top opening of the tea bag, repeated three times. The swelling ratio was calculated using the equation below:

(4) $$SR\;(\%)\;=\;(M_{2}\;-\;M_{1})/M_{1}\;\times \;100$$

#### 2.5.4. Surface Morphology

Micrographs of konjac powders were observed under a JSM-6390LV scanning electron microscope (NTC, Tokyo, Japan) with an accelerating voltage of 10 kV at ×60, ×500 and ×2000 magnifications, and the samples were coated with gold before analysis [28,29].

#### 2.5.5. Bulk Density and Tapped Density

Bulk and tapped density were considered important factors in powder processing and handling. In this regard, the bulk density and tapped density of konjac powders were determined following the methods described by AlYammahi et al. (2023) and Fikry et al. (2021), with minor modifications [30,31].

To determine bulk density, the konjac powders (about 3 g, M) were added into a measuring cylinder (10 mL) and the volume of the powders (V_1_) was recorded; this was repeated three times. The bulk density (Ρ_bulk_) was calculated using the equation below:

(5) $${Ρ}_{bulk}=M/V_{1}$$

The konjac powders (about 3 g, M) were added into a measuring cylinder (10 mL) to determine the tapped density. Then, the samples were shaken vigorously for 3 min and observed until there was no significant change in volume, and the volume V_2_ was recorded. Tapped density (Ρ tap) was calculated using the equation below:

(6) $${Ρ}_{tap}=M/V_{2}$$

#### 2.5.6. Compressible Index and Hausner Ratio

The compressible index (CI) and Hausner ratio (HR) primarily reflect the compressibility and fillability of the material. The better the flowability of the material, the closer the bulk density is, and the lower the CI value. The compressible index was calculated using the equation below [32]:

(7) $$CI=({Ρ}_{tap}-{Ρ}_{bulk})/{Ρ}_{tap}\times 100$$

(8) $$HR={Ρ}_{tap}/{Ρ}_{bulk}$$

#### 2.5.7. Angle of Repose Determination

The repose (α) angle was measured by the method of Huang et al. (2020) and Zhu et al. (2022), with minor modifications [33,34]. The glass funnel was fixed vertically on the desktop and placed under the glass plate of the funnel. The height of the funnel tail end from the glass plate was 5 cm. A certain amount of konjac powders (about 9 g) flowed vertically through the funnel to the glass plate to form a cone. Digital calipers were used to determine the height of the cone H and the bottom of the circular radius, and this was repeated three times. The angle of repose was calculated using the equation below:

(9) α=arc tan H/R

#### 2.5.8. True Density and Porosity Determination

The true density of konjac powders was determined using the liquid displacement method with slight modifications as described by Sandhan et al. (2019) [32,35].

A dry and clean gravity flask was weighed and designated as M_1_. A specific mass of konjac powders labeled M_0_ was introduced into a clean specific gravity flask. Subsequently, anhydrous ethanol was added to the flask, ensuring the volume did not exceed half of its capacity. The flask was then placed in a vacuum-drying oven for 40 min to evacuate the vacuum. Upon removal, the liquid on the flask’s surface was wiped off, and the flask was filled with anhydrous ethanol, with any excess liquid on the surface being removed. The flask was emptied, rinsed, and refilled with anhydrous ethanol, after which the excess liquid was wiped off. The flask was then weighed accurately as M_2_, followed by a precise weighing as M_3_, repeated thrice. The true density was calculated using the equation below:

(10) $${Ρ}_{t}=M_{0}/(M_{3}+M_{0}-M_{2})\times {Ρ}_{w}$$

where Ρ_t_ is the true density of konjac powders at room temperature, and Ρ_w_ is the density of anhydrous ethanol at room temperature.

The porosity was calculated using the equation below:

(11) $$Porosity\left(\%\right)=1-({Ρ}_{bulk}/{Ρ}_{t})\times 100$$

### 2.6. Compressibility Measurement

#### 2.6.1. Tensile Strength (TS)

The TS was measured using the method described by Nwachukwu et al. (2020) and Yu et al. (2020) with minor modifications [36,37,38].

A quantity of uniform-quality konjac powders was weighed, and tablets were formed using a hydraulic tablet press at pressures of 6 MPa, 12 MPa, 18 MPa, and 24 MPa. The tablets were placed in a desiccator for 24 h, after which they were weighed and recorded as M_1_. Subsequently, the thickness and diameter of the tablets were measured using digital calipers and recorded as R and H, respectively. The tablet’s hardness was assessed using a hardness test (Yellow Sea Drug Testing Instrument, Shanghai, China), which was documented as F and repeated three times. The TS was calculated using the equation below:

(12) TS=2F/πRH

#### 2.6.2. Kawakita Equation

The Kawakita equation was used to describe the degree of reduction in powder bed volume as compression pressures increase [39,40]:

(13) P/C=(P/a)+(1/ab)

(14) $$C=(V_{0}-V_{1})/V_{0}$$

V_0_ is the volume of the power bed without compression, and V_1_ is the volume at a given compression pressure. P is the given compression pressure. C is the degree of volume reduction. a and b are the compression parameters. ab is the degree of particle rearrangement.

### 2.7. Tablet Preparation

Tablets with a constant weight of 500 mg were prepared by direct compression of a homogeneous mixture of konjac powders with excipients (microcrystalline cellulose, oligofructose) using a single punch tablet press equipped (Shanghai Tian fan Medicine Machine Manufacture Factory, Shanghai, China) with a 13 mm round punch [41,42].

### 2.8. Tablet Quality

#### 2.8.1. Thickness and Diameter of Tablets

The thickness and diameter of the tablets were measured using the methods described by Arrora with minor modifications [43].

The thickness and diameter of the tablets were measured using vernier calipers (Deli Stationery Group Co., Ltd., Ningbo, China). Six tablets from each batch were randomly selected for measurement.

#### 2.8.2. Weight Variation

The weight variation of tablets was examined according to the method by the Chinese Pharmacopoeia 2020 with slight modifications [44].

Took 20 tablets of the prepared tablets, accurately weighed the total weight using an analytical balance (Beijing Sartorius Scientific Instruments Co., Ltd., Beijing, China), found the average weight of the tablets, then weighed each of the 20 tablets individually and recorded the weights. Then, the weight of each tablet with the average tablet weight (for tablets without assay or Chinese medicine tablets with a marked tablet weight, the weight of each tablet should be compared with the marked tablet weight). No more than 2 tablets might exceed the weight difference limit, and no tablet might exceed the limit by a factor of 1, as shown in Table 2.

#### 2.8.3. Hardness of Tablets

The hardness of tablets was measured using the method described by Shiva et al. with minor modifications [45].

The tablet was placed between the two pressure plates of the automatic hardness tester (Shanghai Huang hai Pharmaceutical Instrument Co., Ltd., Shanghai, China). The pressure was applied along the diameter direction, and the force when the tablet just broke was the tablet’s hardness, which was repeated 6 times. Regarding the hardness of tablets, the Chinese Pharmacopoeia 2020 had not made uniform provisions; generally, it could withstand a pressure of 30–40 N (35–80 N), which was considered qualified.

#### 2.8.4. Friability of Tablets

The difference in tablet weight was examined according to the method by the Chinese Pharmacopoeia 2020 with slight modifications [44].

A number of tablets were taken, so their total weight was 6.5 g or more. The powders on the surface of the tablets were blown off with a hairdryer, accurately weighed, and recorded as M_0_. The weighed tablets were then placed in the cylinder of the friability tester (Tianjin Tianda Tian fa Technology Co., Ltd., Tianjin, China), and the instrument was started. When the instrument stopped rotating, it was removed, and the powders on the tablet’s surface were blown off with a hairdryer, accurately weighed, and recorded as M_1_. The friability of tablets was calculated using the equation below. The friability of the tablets should not exceed 1%, and no broken, cracked, or crushed tablets should be found.

(15) $$Friability=(M_{0}-M_{1})/M_{0}\;*\;100$$

### 2.9. Statistical Analysis

The experimental data were statistically analyzed using IBM SPSS Statistics. After the data were tested for normality and chi-square, the treatment effects of each structure were assessed using the one-way ANOVA method. Waller–Duncan analysis was chosen to compare the means between groups for significance testing, with *p* < 0.05 indicating a significant difference. The results were expressed as mean ± standard deviation, and plots of the experimental data were drawn using Origin 2020 b.

## 3. Results and Discussion

### 3.1. Effect of Moisture Regulation Treatment on Physical Properties of Konjac Powders

#### 3.1.1. The Hydration Properties, Swelling Characteristics, and Particle Size of Konjac Powders

**Figure 1:**
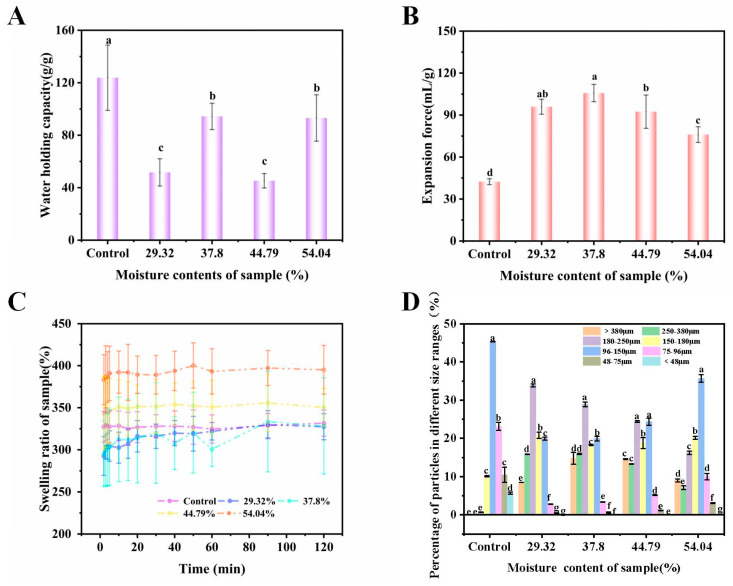
Water-holding capacity (**A**), expansion force (**B**), dissolution curve (**C**), and particle size distribution (**D**) of konjac powders treated with different moisture contents (29.32–54.04%) (different letters a–g indicate significant differences between groups (*p* < 0.05)).

Figure 1 illustrates the alterations in hydration properties, swelling characteristics, and particle size of konjac powders following treatment with varying moisture contents (29.32–54.04%). Compared to the control group, the water-holding capacity of the treatment group decreased significantly. This phenomenon might be attributed to the penetration of water molecules, which promoted the formation of hydrogen bonds between the hydroxyl groups of KGM molecules and those of water molecules, leading to a decrease in the number of hydroxyl groups in the KGM molecules, which had a more pronounced effect on the formation of hydrogen bonds with water molecules, in agreement with the previous report [46]. Furthermore, the structural porosity of konjac powders treated with VFD also rose gradually, decreasing the water retention capacity of the konjac powders [47].

The expansion force and swelling capacity of the VFD group were significantly higher than those of the control group. This could be because the size or number of ice crystals formed during pre-freezing increased with the controlled moisture content, which in turn increased the porosity of the vacuum freeze-drying powder. As a result, water molecules were more likely to enter the pores, and the konjac powders absorbed water and expanded, leading to an increase in the expansion force, consistent with previous reports [48,49]. On the other hand, the expansion force of konjac powders tended to rise and then fall as the controlled moisture content increased. The 37.80% treatment group had a good expansion force, which could be because the original vapor phase destroyed the volume of ice crystals.

Figure 1D illustrates the variation in particle size of konjac powders subjected to different moisture contents (29.32–54.04%). As the regulated moisture content increased, the particle size of konjac powders gradually increased. This phenomenon might be attributed to the absorption of significant amounts of water by konjac powders during moisture regulation, resulting in swelling. Upon freezing, the absorbed water crystallized into ice, occupying space and maintaining structural integrity post-drying, thereby augmenting the volume of konjac powders [50,51]. Furthermore, the increase in moisture content likely resulted in a reduction of crystalline regions, an enhancement of hydrogen bond strength, and the infiltration of water molecules into the entangled cellulose chains, which loosened the polymer structure, ultimately contributing to its swelling and increased particle size [52].

#### 3.1.2. The Powder Properties, True Density, and Porosity of Konjac Powders

Powder properties included properties such as the flowability and compressibility of powders, which had a significant impact on the processing of powders for industrial applications and transport, such as storage in hoppers and silos, transport, and tablet preparation. Poor flowability and compressibility could lead to negative effects, such as a deterioration in the quality of the tablets being prepared and discharged from the silo [53,54]. Powder properties were, therefore, an important reference for using powders in industrial production.

Table 3 shows the powder properties of konjac powders treated with different moisture contents (29.32–54.04%). As the regulated moisture content increased, the bulk and tapped densities of konjac powders gradually decreased, and the difference between treatment groups was significant. The tapped density indicated the porosity of the powder, which exhibited an inverse relationship [55,56]. This finding suggested that the moisture conditioning treatment enhanced the porosity of konjac powders, with an increase corresponding to higher moisture content, as corroborated by the porosity measurements presented in Table 4. Hausner index and Carr’s index indicated powders’ filling characteristics, while the repose angle demonstrated their flowability. Table 3 revealed that the Hausner index was less than 1.2, and Carr’s index was less than 15% for all treatment groups. On the other hand, the angle of repose was significantly reduced in all treatment groups except the 54.04% moisture treatment group, which showed no significant change. This indicated that the moisture modification combined with vacuum freeze-drying treatment improved the fillability of konjac powders, while flowability was less affected, and powder properties were improved.

#### 3.1.3. The Microscopic Morphology of Konjac Powders

**Figure 2:**
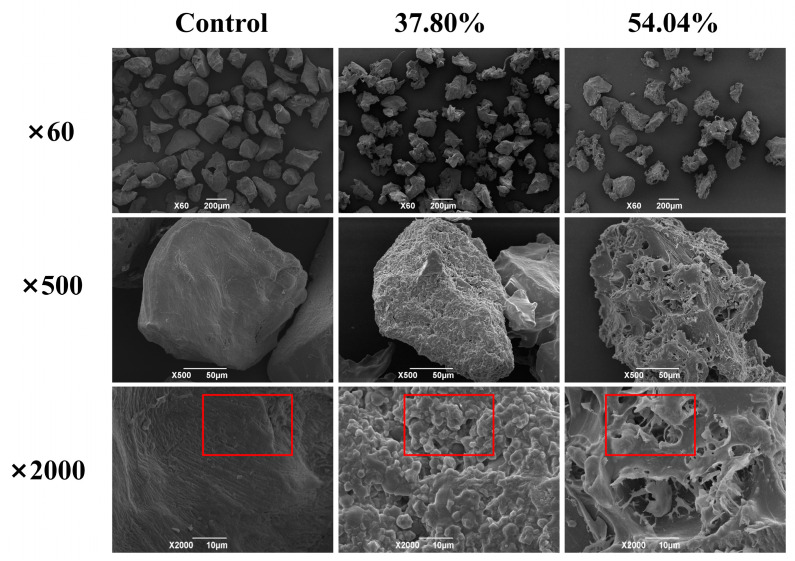
Microscopic morphology of konjac powders treated with different moisture contents (37.80–54.04%).

This study involved selecting three groups of konjac powders with varying moisture levels—low, medium, and high—for SEM measurements to illustrate the impact of moisture control on the micro-morphology of konjac powders. Figure 2 illustrates the microscopic morphology of the moisture-conditioned konjac powders. The particles in the control group exhibited a regular shape, characterized by a smooth surface devoid of edges. Further magnification revealed the surface was smoother and denser, lacking any discernible pores. The particles in the treatment group exhibited irregular shapes characterized by distinct angles, a rough surface, and a loose, porous structure. Additionally, the size of pores increased with higher regulated moisture content. The observed variations could be attributed to differences in moisture content among the particles, the formation of distinct ice crystals during the pre-freezing phase, and the resultant pore structures following vacuum freeze-drying, where higher moisture samples yield larger ice crystals and, consequently, larger pores after sublimation.

### 3.2. Effect of Single Drying Methods on Physical Properties of Konjac Powders

#### 3.2.1. The Hydration Properties and Swelling Characteristics of Konjac Powders

**Figure 3:**
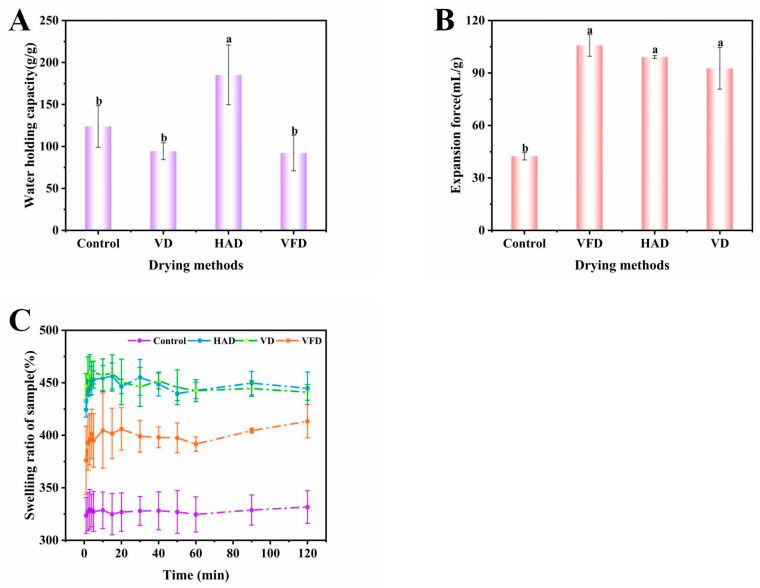
Water-holding capacity (**A**), expansion force (**B**), and dissolution curve (**C**) of konjac powders treated with 37.80% moisture modification combined with three drying methods (different letters a,b indicate significant differences between groups (*p* < 0.05)).

Figure 3 shows the changes in hydration properties and swelling characteristics of konjac powders treated with 37.80% moisture modification combined with different drying methods. Compared to the control group, the water-holding capacity of the HAD treatment group was significantly increased. At the same time, there was no significant change in the VFD and VD treatment groups. All three drying methods significantly increased konjac powders’ expansion force and swelling capacity. This might be due to the differences in the effects of different drying methods on the microstructure of konjac powders. The surface exhibited unevenness following HAD treatment, resulting in increased structural density [57]. Post-vacuum drying, the surface exhibited smoothness but also ruptures [58]. In contrast, following vacuum freeze-drying, the surface appeared rough, loose, and porous. Kapoor et al. (2022) reported analogous observations regarding VFD powders [59]. The loose and porous microstructure facilitated water entry and enhanced expansion force and swelling capacity; however, it posed challenges in water retention, potentially leading to a decline in water-holding capacity.

#### 3.2.2. The Powder Properties, True Density, and Porosity of Konjac Powders

Table 5 shows the powder properties of konjac powders treated with 37.80% moisture modification combined with different drying methods (HAD, VFD, and VD). Compared to the control group, the tapped density of konjac powders was significantly reduced after all three drying treatments. This finding indicated that the treatment increased the porosity of konjac powders, which was consistent with the porosity measurements in Table 6. The Hausner and Carr indices of konjac powders treated by vacuum freeze-drying were significantly lower than those of the other two drying methods, indicating better compressibility in the vacuum freeze-drying treated group. On the other hand, the angle of repose did not change significantly, indicating that the drying method had less effect on the flowability of the konjac powders. In conclusion, the powder properties of konjac powders treated by vacuum freeze-drying were optimal, which was related to their loose and porous microstructure, in agreement with the results reported in the literature [60,61].

#### 3.2.3. The Microscopic Morphology of Konjac Powders

**Figure 4:**
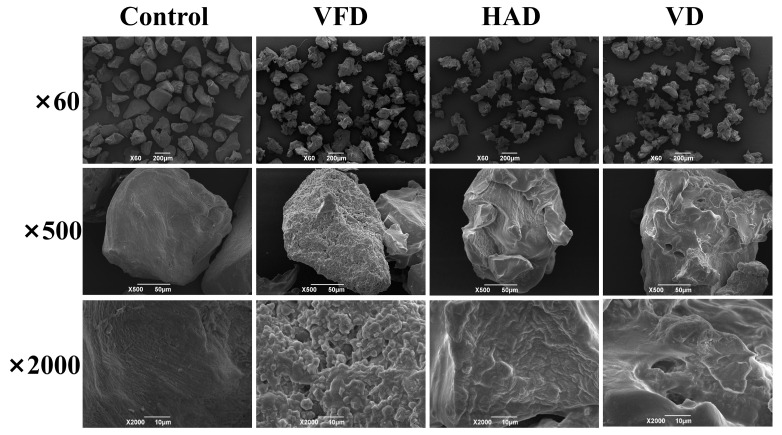
Microscopic morphology of konjac powders treated with 37.80% moisture modification combined with three drying methods.

Figure 4 presents the results of the microscopic morphology analysis of konjac powders subjected to various drying methods. Compared to the control group, the surface of the konjac powders treated by the three drying methods exhibited irregularities. Further magnification revealed significant variations in the surface morphology of the konjac powders across the different drying methods. The particle surfaces after VFD treatment exhibited a loose and porous morphology with a rough texture. In contrast, the HAD treatment resulted in a dense layer structure with minimal wrinkling. The VD treatment produced uneven particle surfaces characterized by a limited number of pore structures, consistent with previous reports [62]. The variation in appearance resulted from the differing drying rates and moisture removal associated with the various drying methods.

### 3.3. Effect of Different Combined Drying Methods on Physical Properties of Konjac Powders

#### 3.3.1. The Hydration Properties and Swelling Characteristics of Konjac Powders

**Figure 5:**
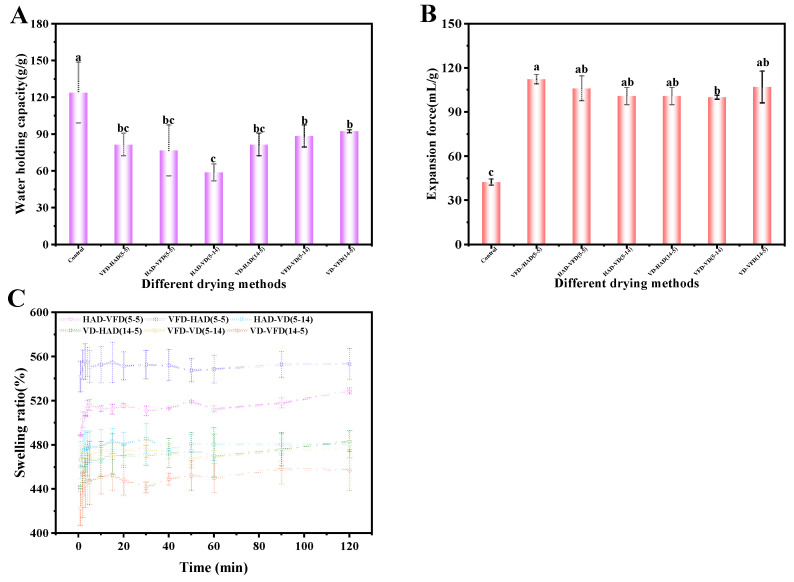
Water-holding capacity (**A**), expansion force (**B**), and dissolution curves (**C**) of konjac powders treated with six combinations of drying methods at 37.80% moisture content (different letters a–c indicate significant differences between groups (*p* < 0.05)).

Based on the previously discussed modification methods, the konjac powders did not achieve optimal hydration and swelling properties. To address the limitations of a single drying method, this experiment selected a combination of drying treatments to overcome these shortcomings [63,64]. The goal was to evaluate the effects of these combined treatments on the hydration and swelling properties of konjac powders. The changes in hydration and swelling properties of konjac powders treated with different combinations of drying methods at 37.80% moisture content are shown in Figure 5. Compared to the control group, the treatment groups exhibited a significant increase in expansion force. However, the water-holding capacity decreased significantly, and the dissolution ratio increased significantly in all treatment groups, except for the VD-VFD combination. No significant differences between the treatment groups were observed in the hydration and swelling characteristics. Moreover, the increase in dissolution rate might pose challenges in its industrial application, potentially leading to production difficulties.

The results indicated that the order of the combined drying methods had a minimal effect on the hydration and swelling characteristics of KGM powders, which was inconsistent with previous studies [64]. This discrepancy could be attributed to differences in the processed polysaccharides and the potentially inappropriate drying times selected for each drying stage. Consequently, the combined drying treatment was as effective as a single drying method. Therefore, further investigation is required to explore the influence of the moisture transition point on the physical properties of KGM powders.

#### 3.3.2. The Powder Properties, True Density, and Porosity of Konjac Powders

The results of powder properties determination of konjac powders treated with different combinations of drying methods at 37.80% moisture content are presented in Table 7. The bulk density and tapped density were significantly lower in all treatment groups than in the control group, while there was no significant difference between treatment groups. The reduction in tapped density and the rise in porosity suggested that the treatments altered the pore structure of konjac powders, aligning with the porosity measurements presented in Table 8.

No significant changes were observed in the Hausner index, Carr’s index, and angle of repose, indicating that the drying method minimally influenced the fillability and flowability of konjac powders. In summary, the powder properties of konjac powders exhibited minimal variation due to the combination of drying methods. This consistency could be attributed to the stable initial moisture content before drying and the moisture content at the endpoint. Moisture content significantly influenced the flowability of dry powders [65], resulting in comparable alterations in the micro-morphology of konjac powders following treatment with various drying method combinations.

#### 3.3.3. The Microscopic Morphology of Konjac Powders

**Figure 6:**
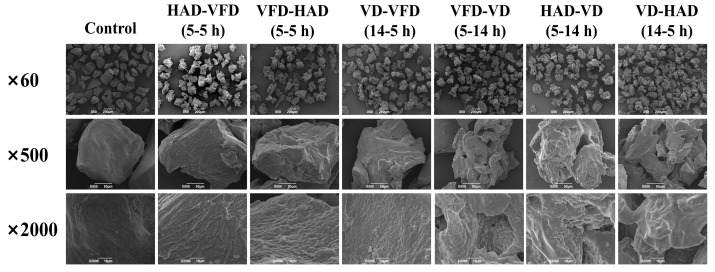
Microscopic morphology of konjac powders treated with six combinations of drying methods at 37.80% moisture content.

The results of the micro-morphological analysis of konjac powders treated with different combinations of drying methods at 37.80% moisture content are presented in Figure 6. Figure 6 illustrates that, compared to the control group, the microscopic morphology of konjac powders treated with different combined drying methods exhibited significant changes. Notable increases in roughness and folds were observed, along with the formation of a distinct pore structure. The particles appeared irregular, angular, and prominently rough. Moreover, significant differences were observed between the treatment groups. The HAD-VFD (5-5 h), VFD-HAD (5-5 h), and VD-VFD (14-5 h) treatment groups exhibited a dense laminar structure. The VFD-VD (5-14 h), HAD-VD (5-14 h), and VD-HAD (5-5 h) treatment groups exhibited a notably rougher and looser pore structure. The observed difference might result from the combination and sequence of various drying methods, consistent with previous literature [64].

### 3.4. Effect of Moisture Transition Point on Physical Properties of Konjac Powders

#### 3.4.1. The Hydration Properties and Swelling Properties of Konjac Powders

**Figure 7:**
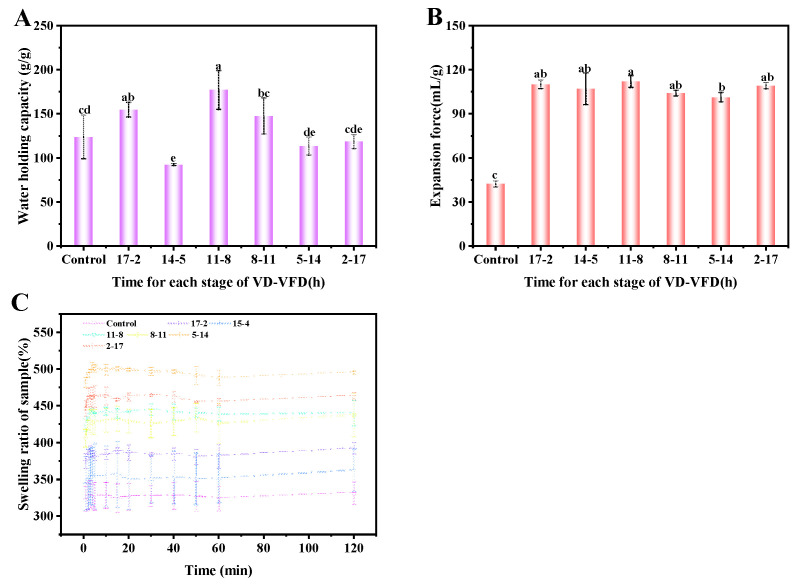
Water-holding capacity (**A**), expansion force (**B**), and dissolution curve (**C**) of konjac powders treated with VD-VFD at different moisture transition points (different letters a–e indicate significant differences between groups (*p* < 0.05)).

The moisture conversion point (the sample’s moisture content after pre-drying in the combined drying process) was another significant factor influencing the combined drying efficiency [66]. The changes in the hydration properties and swelling characteristics of konjac powders treated with VD-VFD at different moisture transition points are shown in Figure 7. As indicated in Figure 7, there was a significant difference in water-holding capacity among the groups, with a general trend of decreasing water-holding capacity as the vacuum freeze-drying time increased. This trend could be attributed to the rise in the moisture conversion point. Pre-freezing materials with high moisture content formed larger ice crystals at the same temperature, compromising the material’s original structure. This process increased the voids formed by the sublimation of the ice crystals, thereby reducing the water retention capacity. The treatment group subjected to 11 h of vacuum drying followed by 8 h of vacuum freeze-drying exhibited the highest water-holding capacity.

Compared to the control group, the expansion force of konjac powders treated with VD-VFD at various moisture conversion points significantly increased. However, no notable differences were observed among the different treatment groups. The swelling curves of konjac powders subjected to different moisture conversion points are shown in Figure 7C. As indicated in the figure, there were significant variations in the dissolution rates among the treatment groups, with a general trend showing that the dissolution rate of the samples increased as the freeze-drying time was extended. The advancement of the moisture transition point due to freeze-drying resulted in an enhanced pore structure [67], facilitating water ingress. It improved both the konjac powders’ expansion force and swelling capacity. In conclusion, the water-holding capacity and expansion force of the VD-VFD (17-2 h) treatment group were significantly better than those of the control group. Although the swelling properties did not significantly improve, the comprehensive optimization of hydration and swelling properties was achieved, meeting the application requirements for konjac powders with high water-holding capacity and expansion force.

#### 3.4.2. The Powder Properties, True Density, and Porosity of Konjac Powders

Table 9 shows the powder properties of konjac powders treated with VD-VFD at different moisture transition points. As shown in Table 9, the tapped density and bulk density of konjac powders dried in different moisture conversion point treatments were significantly lower than those of the control. Still, there was no significant difference between treatment groups. The porosity of treatment groups increased, consistent with the measurements shown in Table 10. While the Hausner index, Carr’s index and angle of repose were not significantly different from the control group. The results of the powder properties measurements showed that the changing of the moisture transition point had little effect on the powder properties of the samples.

#### 3.4.3. The Microscopic Morphology of Konjac Powders

**Figure 8:**
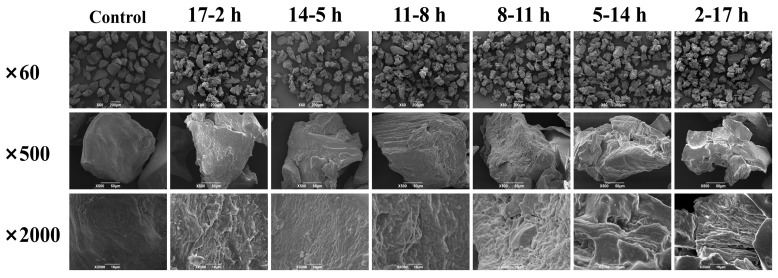
Microscopic morphology of konjac powders treated with VD-VFD at different moisture transition points.

Figure 8 reveals the microscopic morphology of konjac powders treated with VD-VFD at different moisture conversion points. The microscopic morphology of konjac powders changed significantly after treatment at different moisture conversion points. Compared to the control group, particles in all treatment groups were significantly angular, rough, and irregular, with increased surface wrinkles. In the VD treatment group, the degree of looseness gradually increased as the vacuum drying time was reduced. When the vacuum drying time was 8 h and below, an obvious pore structure could be observed on the surface of konjac powders. This was because as the vacuum time was shortened (the moisture conversion point was advanced), the moisture content of konjac powders gradually increased, the ice crystals formed after pre-freezing became larger or more numerous, and the pore structure formed after vacuum freeze-drying treatment also increased, which was consistent with previous literature [68].

### 3.5. Compressibility Characterization

Powder properties and porosity measurements showed an increase in the porosity of treated konjac powders; the larger the porosity, the better the compressibility [69], indicating that the compressibility of konjac powders was improved after treatment. Therefore, the Kawakita equation and the TS pressure curve were chosen to characterize the compressibility of konjac powders.

#### 3.5.1. Kawakita Equation for Untreated and Various Modification Konjac Powders

The results of fitting the Kawakita equation for konjac powders with different moisture content treatments are exposed in Table 11. Compared to the control group, 37.80% of the treatment group’s a value and 1/b value increased, which indicated an increase in compressibility and a decrease in the plasticity of konjac powders. There was no significant change in a and 1/b values for the 54.04% treatment group, indicating that the change in compressibility and plasticity of konjac powder treated with 54.04% moisture treatment combined with vacuum freeze-drying was less. However, according to the available literature and other properties determined above, the compressibility of this treatment group was improved due to the increase in structural porosity. There was a contradiction between the results; therefore, the compressibility needed further evaluation in conjunction with the TS pressure curve.

Table 12 shows the results of fitting the Kawakita equation for konjac powder treated with different drying methods. Compared with the control group, the a value of the treated group increased, and the 1/b value of the VD and VFD treated groups increased, indicating that the three drying methods improved the compressive properties of konjac powder, and the plasticity of konjac powders decreased in the VD and VFD treated groups. The 1/b value decreased in the HAD-treated group, and the powder’s plasticity increased, adversely affecting its use as a raw material in the manufacture of tablets. This difference might be due to the different drying methods, which had different effects on the microscopic morphology of konjac powder, affecting the powder’s tablet-forming properties, consistent with the previously reported literature [70]. In conclusion, the tablet-forming properties increased in the VD and VFD treatment groups, but further analysis was required in conjunction with the TS measurements.

Table 13 shows that the 1/b values of all treatment groups were significantly increased, indicating that the plasticity of each treatment group was reduced compared to the control group, which was favorable for using konjac powders as a raw auxiliary material for the preparation of tablets. The value decreased in all the treatment groups except the HAD-VFD treatment group, which increased. This indicated that the rest of the combined drying treatments, except for the HAD-VFD treatment, negatively affected the compressibility of konjac powders. Therefore, it was necessary to further analyze the effects of different combined drying methods on the flake formation properties of konjac powder in conjunction with the TS measurement results.

Table 14 presents the Kawakita equation for konjac powders subjected to different moisture transition points. Compared to the control group, the 1/b value increased, while plasticity decreased in the treatment group. The a-value exhibited an initial increase followed by a decrease as vacuum drying time was reduced. The treatment group with a duration of 14-5 h demonstrated the highest a-values. This might be due to the difference in compression properties due to the change in porosity formed during vacuum freeze-drying of samples entering the pre-freezing stage with different moisture contents, consistent with previously reported literature [70]. However, further analysis was required in conjunction with the results of the TS measurements

#### 3.5.2. TS–Pressure Change Curve for Untreated and Various Modification Konjac Powders

**Figure 9:**
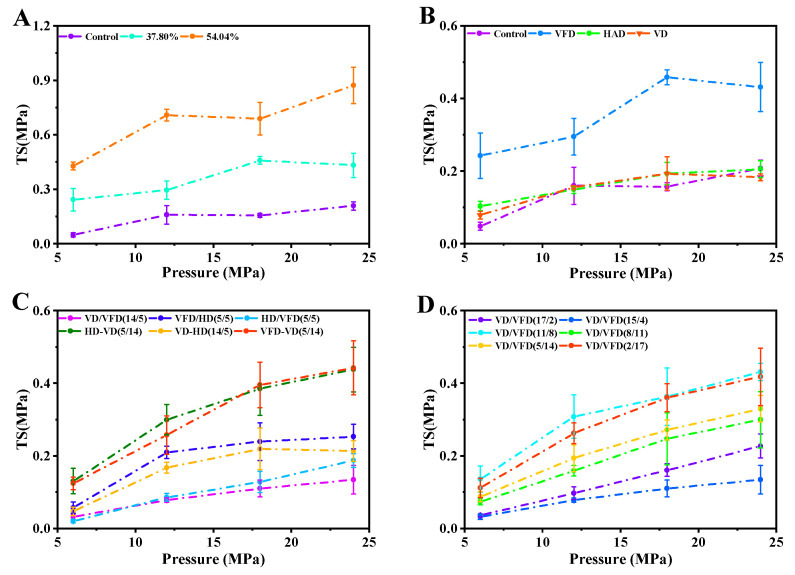
TS–pressure change curves of tablets prepared from konjac powders with different moisture content treatments (29.32–54.04%) (**A**), 37.80% moisture modification combined with three drying treatments (**B**), six different combinations of drying methods at 37.80% moisture content (**C**), and different moisture transition points treatments (**D**).

Figure 9 illustrates the TS (tensile strength)–pressure change curve of tablets formulated from konjac powders under four treatments. Compared to the control group, at the same pressure, the TS values of konjac powders treated with different moisture contents were significantly higher than those of the control group and increased with the increased regulated moisture content.

The TS values of konjac powders subjected to different drying methods exhibited significant variation. The TS in the VD treatment group showed a notable increase, whereas the other two drying methods did not differ significantly from the control group. The TS of the VFD treatment group was distinguished from the other two drying methods by its loose and porous microstructure.

The results presented in Figure 9C demonstrated that the TS values of konjac powders were influenced by the combination and sequence of drying methods, showing a significant increase in the TS values for the VFD-VD and HAD-VD treatment groups. Figure 9D illustrates significant differences in the TS of konjac powders treated at different moisture transition points, with the highest TS values observed for konjac powders treated at the time ratios of VD-VFD (11-8 h) and VD-VFD (2-17 h), corresponding to a total drying time of 19 h. The TS of the 54.04% moisture treatment group was significantly higher than that of the other sample groups at the same pressure, indicating optimal compression of konjac powders [71]. The porosity of the powder was significantly associated with its compactibility; higher porosity correlated with improved compactibility of the sample. The 54.04% moisture treatment group exhibited high porosity and a rough microstructure, aligning with the findings from SEM, powder properties, and porosity analyses. Analysis of the measurements indicated that the treatment group with 54.04% moisture exhibited the highest level of compactibility.

### 3.6. Tablet Quality of Tablets Prepared from Untreated and Treated Konjac Powders

The following tablet formulations were determined in the preliminary experiments (Appendix A):

75% konjac powders +15% MCC (microcrystalline cellulose) +10% OFOS. The quality of tablets formulated using commercial konjac powders and treated konjac powders in this study, utilizing identical formulation and pressure, was presented in Table 15. According to the stipulations of the Chinese Pharmacopoeia [44], there was a significant difference in the quality of the tablets prepared between the two groups. Tablets prepared using commercially available konjac powders were of poor quality. In contrast, the tablets produced from the processing of konjac powders were of good quality and complied with the requirements of the Pharmacopoeia. The results indicated that the tablets manufactured from treated konjac powders exhibited a denser structure, reflecting the better compressibility of treated konjac powders.

### 3.7. Research Limitations and Future Prospects

In this study, limited moisture modification combined with drying methods was used to treat konjac powders, and the effects of the treatments on the hydration characteristics and powder properties of konjac powders were investigated in detail for the first time. However, the various health benefits of konjac powders were closely related to their physico-chemical properties, such as hydration, viscosity, and fermentation properties. In addition, konjac powders’ rheological and gelling properties were significant for their industrial application. Therefore, several other aspects needed to be investigated to ensure that the konjac powders produced in this study could be widely used in industrial production as a novel excipient. Therefore, this study needed to characterize further the changes in viscosity, rheological properties, gel properties, and fermentation properties of each sample group using experimental methods such as the rheometer test to comprehensively evaluate the effects of the modification method on the physico-chemical properties of konjac powders, and to provide more comprehensive and detailed theoretical data support for its use as a new type of excipient in industrial production.

## 4. Conclusions

In conclusion, the limited moisture modification combined with drying methods proved to be an effective control strategy to improve several physical properties of konjac powders after treatment. Compared to the control group, significant changes in micromorphology were observed in the treatment groups, and the degree of looseness and powder compressibility were improved to varying degrees. The hydration characteristics of the VD17 h-VFD2 h treatment group increased significantly. The 54.04% moisture adjustment combined with the VFD treatment group showed the best compressibility, which could not only meet the production requirements of direct compression of powder but also increase the proportion of konjac powders in tablets under the premise of meeting the quality of tablets. The konjac powders processed in this study were suitable for the application scenarios of high water-holding capacity, high expansion force, and powder direct compression tablets, which expanded the application field of konjac powders. This research provided a green and simple design strategy and technical parameter references for diversified modification of the physical properties of konjac powders. However, it is necessary to further investigate the effects of modification methods on the physico-chemical properties of konjac powders, such as rheology and gelation, and to comprehensively evaluate the impact of modification methods on the physico-chemical properties of konjac powders to provide comprehensive theoretical guidance for their diverse industrial applications.

## Figures and Tables

**Table 1:** Drying parameters of different combined drying methods.

Combined Drying Methods	Drying Temperature	Drying Time
VFD-VD (vacuum freeze-drying combined with vacuum drying)	−40–50 °C	5 h–14 h
VD-VFD (vacuum drying combined with vacuum freeze-drying)	50–−40 °C	14 h–5 h
VD-HAD (vacuum drying combined with hot air drying)	50–50 °C	14 h–5 h
HAD-VD (hot air drying combined with vacuum drying)	50–50 °C	5 h–14 h
HAD-VFD (hot air drying combined with vacuum freeze-drying)	50–−40 °C	5 h–5 h
VFD-HAD (vacuum freeze-drying combined with hot air drying)	−40–50 °C	5 h–5 h

**Table 2:** Criteria for weight variation according to the Chinese Pharmacopoeia.

Average Film Weight or Marked Film Weight	Weight Deviation Limits
Less than 0.3 g	±7.5%
0.3 g and above	±5.0%

**Table 3:** Powder properties of konjac powders treated with different moisture contents (29.32–54.04%) (different letters a–d indicate significant differences between groups (*p* < 0.05)).

Sample	Bulk Density (g/cm^3^)	Tapped Density (g/cm^3^)	Hausner Index	Carr Index (%)	Angle of Repose (°)
Control	0.64 ± 0.02 ^a^	0.72 ± 0.01 ^a^	1.13 ± 0.02 ^b^	11.68 ± 1.15 ^a^	31.94 ± 1.48 ^ab^
29.32%	0.55 ± 0.00 ^b^	0.60 ± 0.00 ^b^	1.08 ± 0.07 ^c^	7.41 ± 0.00 ^b^	25.88 ± 3.22 ^b^
37.80%	0.55 ± 0.14 ^b^	0.59 ± 0.02 ^b^	1.08 ± 0.00 ^c^	7.28 ± 0.19 ^b^	26.35 ± 4.29 ^b^
44.79%	0.49 ± 0.12 ^c^	0.54 ± 0.00 ^c^	1.09 ± 0.03 ^c^	8.17 ± 2.13 ^b^	28.07 ± 1.51 ^b^
54.04%	0.44 ± 0.01 ^d^	0.51 ± 0.01 ^d^	1.17 ± 0.15 ^a^	14.50 ± 0.30 ^a^	35.79 ± 6.50 ^a^

**Table 4:** True density and porosity of konjac powders treated with different moisture contents (37.80–54.04%) (different letters a–c indicate significant differences between groups (*p* < 0.05)).

Moisture Contents	Ρ_t_ (True Density)	∈ (Porosity %)
Control	1.628 ± 0.052 ^a^	59.181 ± 2.812 ^c^
37.80%	1.732 ± 0.165 ^a^	68.631 ± 0.663 ^b^
54.04%	1.827 ± 0.179 ^a^	75.539 ± 1.144 ^a^

**Table 5:** Powder properties of konjac powders treated with 37.80% moisture modification combined with three drying methods (different letters a–c indicate significant differences between groups (*p* < 0.05)).

Drying Methods	Bulk Density (g/cm^3^)	Tapped Density (g/cm^3^)	Hausner Index	Carr Index (%)	Angle of Repose (°)
Control	0.64 ± 0.02 ^a^	0.72 ± 0.01 ^a^	1.13 ± 0.02 ^c^	11.68 ± 1.15 ^b^	31.94 ± 1.48 ^a^
VFD	0.55 ± 0.14 ^a^	0.59 ± 0.02 ^b^	1.08 ± 0.00 ^c^	7.28 ± 0.19 ^c^	26.35 ± 4.29 ^a^
HAD	0.49 ± 0.01 ^b^	0.56 ± 0.01 ^c^	1.15 ± 0.00 ^ab^	12.90 ± 0.00 ^b^	31.94 ± 1.48 ^a^
VD	0.47 ± 0.01 ^b^	0.57 ± 0.01 ^bc^	1.20 ± 0.05 ^a^	18.75 ± 0.00 ^a^	26.35 ± 4.29 ^a^

**Table 6:** True density and porosity of konjac powders treated with 37.80% moisture modification combined with three drying methods (different letters a–d indicate significant differences between groups (*p* < 0.05)).

Drying Methods	Ρ_t_ (True Density)	∈ (Porosity %)
Control	1.628 ± 0.052 ^a^	59.181 ± 2.812 ^d^
VFD	1.732 ± 0.165 ^a^	68.631 ± 0.663 ^c^
HAD	1.722 ± 0.098 ^a^	71.577 ± 0.537 ^b^
VD	1.909 ± 0.307 ^a^	75.179 ± 0.456 ^a^

**Table 7:** Powder properties of konjac powders treated with six combinations of drying methods at 37.80% moisture content (different letters a,b indicate significant differences between groups (*p* < 0.05)).

Combined Drying Method	Individual Drying Times(h-h)	Bulk Density (g/cm^3^)	Tapped Density (g/cm^3^)	Hausner Index	Carr Index (%)	Angle of Repose (°)
Control	/	0.64 ± 0.02 ^a^	0.72 ± 0.01 ^a^	1.13 ± 0.02 ^a^	11.68 ± 1.15 ^a^	31.94 ± 1.48 ^a^
HAD-VFD	5-5	0.52 ± 0.02 ^b^	0.59 ± 0.01 ^b^	1.13 ± 0.06 ^a^	11.39 ± 4.85 ^a^	31.32 ± 3.10 ^a^
VFD-HAD	5-5	0.50 ± 0.00 ^b^	0.54 ± 0.03 ^b^	1.10 ± 0.05 ^a^	8.82 ± 4.24 ^a^	31.19 ± 3.35 ^a^
HAD-VD	5-14	0.51 ± 0.02 ^b^	0.58 ± 0.02 ^b^	1.13 ± 0.02 ^a^	11.35 ± 1.76 ^a^	29.18 ± 6.83 ^a^
VD-HAD	14-5	0.51 ± 0.03 ^b^	0.56 ± 0.02 ^b^	1.10 ± 0.02 ^a^	9.06 ± 1.69 ^a^	30.93 ± 0.61 ^a^
VD-VFD	14-5	0.53 ± 0.01 ^b^	0.59 ± 0.01 ^b^	1.11 ± 0.05 ^a^	9.36 ± 3.84 ^a^	26.56 ± 4.69 ^a^
VFD-VD	5-14	0.52 ± 0.03 ^b^	0.58 ± 0.02 ^b^	1.10 ± 0.02 ^a^	9.25 ± 1.61 ^a^	32.33 ± 2.95 ^a^

**Table 8:** True density and porosity of konjac powders treated with six combinations of drying methods at 37.80% moisture content (different letters a–c indicate significant differences between groups (*p* < 0.05)).

Combined Drying Methods	Individual Drying Times (h-h)	Ρ_t_ (True Density)	∈ (Porosity %)
Control	/	1.628 ± 0.000 ^ab^	59.181 ± 2.812 ^c^
HAD-VFD	5-5	1.559 ± 0.047 ^b^	66.801 ± 0.235 ^ab^
VFD-HAD	5-5	1.582 ± 0.004 ^b^	68.909 ± 0.088 ^b^
HAD-VD	5-14	1.560 ± 0.013 ^b^	67.176 ± 1.053 ^ab^
VD-HAD	14-5	1.543 ± 0.043 ^b^	67.394 ± 1.488 ^ab^
VD-VFD	14-5	1.955 ± 0.491 ^a^	72.054 ± 6.956 ^a^
VFD-VD	5-14	1.631 ± 0.200 ^ab^	67.311 ± 2.413 ^ab^

**Table 9:** Powder properties of konjac powders treated with VD-VFD at different moisture transition points (controlled total drying time of 19 h; different letters a–d indicate significant differences between groups (*p* < 0.05)).

Combined Drying Methods (Stage 1-Stage 2)	Individual Drying Times (h)	Bulk Density (g/cm^3^)	Tapped Density (g/cm^3^)	Hausner Index	Carr Index (%)	Angle of Repose (°)
Control	/	0.64 ± 0.02 ^a^	0.72 ± 0.01 ^a^	1.13 ± 0.02 ^ab^	11.68 ± 1.15 ^bc^	31.94 ± 1.48 ^ab^
VD-VFD	17-2	0.50 ± 0.01 ^cd^	0.59 ± 0.03 ^bc^	1.19 ± 0.05 ^a^	15.95 ± 3.88 ^a^	35.65 ± 3.76 ^a^
VD-VFD	14-5	0.53 ± 0.01 ^bc^	0.59 ± 0.01 ^bc^	1.11 ± 0.05 ^b^	9.36 ± 3.84 ^c^	26.56 ± 4.69 ^b^
VD-VFD	11-8	0.48 ± 0.02 ^d^	0.56 ± 0.02 ^c^	1.16 ± 0.06 ^ab^	13.73 ± 4.27 ^b^	35.09 ± 0.59 ^a^
VD-VFD	8-11	0.51 ± 0.01 ^bcd^	0.58 ± 0.02 ^c^	1.14 ± 0.03 ^ab^	12.38 ± 2.07 ^bc^	33.50 ± 0.96 ^a^
VD-VFD	5-14	0.52 ± 0.00 ^bcd^	0.59 ± 0.01 ^bc^	1.13 ± 0.03 ^ab^	11.49 ± 1.99 ^bc^	33.12 ± 3.64 ^a^
VD-VFD	2-17	0.54 ± 0.01 ^b^	0.62 ± 0.01 ^b^	1.14 ± 0.03 ^ab^	12.04 ± 1.96 ^bc^	31.88 ± 2.93 ^ab^

**Table 10:** True density and porosity of konjac powders treated with VD-VFD at different moisture transition points (controlled total drying time of 19 h; different letters a–d indicate significant differences between groups (*p* < 0.05)).

Combined Drying Methods (Stage 1-Stage 2)	Individual Drying Times (h)	Ρ_t_ (True Density)	∈ (Porosity %)
Control	/	1.628 ± 0.000 ^a^	59.181 ± 2.812 ^c^
VD-VFD	17-2	1.633 ± 0.044 ^a^	69.710 ± 0.290 ^a^
VD-VFD	14-5	1.723 ± 0.219 ^a^	69.259 ± 0.612 ^a^
VD-VFD	11-8	1.614 ± 0.032 ^a^	70.281 ± 1.428 ^a^
VD-VFD	8-11	1.377 ± 0.066 ^b^	63.262 ± 0.724 ^b^
VD-VFD	5-14	1.647 ± 0.016 ^a^	68.582 ± 0.006 ^a^
VD-VFD	2-17	1.258 ± 0.027 ^b^	56.866 ± 0.911 ^d^

**Table 11:** Kawakita equation fitting parameters of konjac powders treated with different moisture contents (37.80–54.04%).

Moisture Contents	a	1/b	ab	R^2^
Control	0.673	0.096	7.012	0.999
37.80%	0.815	0.148	5.490	0.999
54.04%	0.636	0.058	10.933	0.999

**Table 12:** Kawakita equation fitting parameters of konjac powders treated with 37.80% moisture modification combined with three drying methods.

Drying Methods	a	1/b	ab	R^2^
Control	0.667	0.042	15.699	0.999
VFD	0.810	0.111	7.309	0.999
HAD	2.498	0.029	86.281	0.999
VD	2.549	0.097	26.364	0.999

**Table 13:** Kawakita equation fitting parameters of konjac powders treated with six combinations of drying methods at 37.80% moisture content.

Combined Drying Methods	Individual Drying Times (h/h)	a	1/b	ab	R^2^
Control	/	0.670	0.096	7.012	0.999
HAD/VFD	5/5	0.910	0.470	1.940	0.998
VFD/HAD	5/5	0.010	0.540	0.020	0.994
HAD/VD	5/14	0.540	0.480	1.130	0.994
VD/HAD	14/5	0.120	0.610	0.200	0.998
VD/VFD	14/5	0.450	0.510	0.880	0.999
VFD/VD	5/14	0.580	0.560	1.040	0.999

**Table 14:** Kawakita equation fitting parameters for konjac powders treated with VD-VFD at different moisture transition points (total drying time of 19 h).

Combined Drying Methods	Individual Drying Times (h/h)	a	1/b	ab	R^2^
VD/VFD	17:2	0.667	0.580	1.160	0.999
VD/VFD	14:5	0.910	0.470	1.940	0.998
VD/VFD	11:8	0.657	0.510	1.290	0.999
VD/VFD	8:11	0.801	0.430	1.870	0.999
VD/VFD	5:14	0.334	0.570	0.580	0.999
VD/VFD	2:17	0.323	0.560	0.570	0.999

**Table 15:** Quality of tablets prepared from untreated/treated konjac powders (different letters a,b indicate significant differences between groups (*p* < 0.05)).

Sample	Weight Variation	Hardness (N)	Brittleness (%)
Control	Does not meet the requirements	29.52 ± 2.73 ^b^	>1% with fragmented, lobes
54.04%	Meet the requirements	40.37 ± 3.00 ^a^	0.79%

Note: Different lowercase letters in the same column indicate significant differences between groups (*p* < 0.05).

## Data Availability

The original contributions presented in this study are included in the article. Further inquiries can be directed to the corresponding author.

## Appendix A

In the experiments, it was found that the tablets prepared with konjac powders alone as the compression material could not become shaped; this was mainly due to the fact that konjac powders, as a fibrous raw material, had poor inter-particle adhesion. Considering the problem of tablet forming, it was decided to introduce excipients to ensure that the tablets could be formed.

MCC had good flowability and compactibility and was widely used as a filler, binder, or disintegrant in the direct compression of powders. Thus, MCC was selected as an excipient for tablet formulation, and the effect of its addition on tablet shape and hardness was investigated. The results are shown in Appendix A Table A1, the tablets were shaped after MCC addition, and the tablet hardness increased significantly with increasing addition.

**Table A1:** Hardness of tablets prepared with different MCC additions.

MCC Additions	Tablet Hardness (N)
10%	15.45 ± 0.10 ^c^
30%	23.89 ± 1.90 ^b^
50%	56.77 ± 3.01 ^a^

Note: Different lowercase letters in the same column indicate significant differences between groups (*p* < 0.05).

Although the addition of MCC could shape the tablets, it was necessary to add a large amount of MCC to make the tablets meet the requirements of the Chinese Pharmacopoeia. In order to increase the percentage of konjac powders in the tablets as much as possible, oligosaccharides were chosen as another excipient in this experiment. This was because oligosaccharides have strong adhesive properties, which can increase the hardness of tablets, make the surface of tablets smooth, and further improve the quality of tablets. And oligosaccharides are not digested and absorbed in the gastrointestinal tract but are utilized and broken down by intestinal micro-organisms. Like KGM, they have prebiotic activity and enhance the active ingredients in tablets. However, oligosaccharides are highly hygroscopic, and excessive addition could lead to quality deterioration during storage. Therefore, in this experiment, a 10% addition was determined and used to study the influence of the types of oligosaccharides on the hardness of tablets. The results are shown in Appendix A Table A2; under the condition of 15% MCC addition, the tablet prepared with the addition of oligofructose had the highest hardness. Therefore, the formulation of the tablet was determined as follows: 75% konjac powders + 15%MCC + 10%OFOS.

**Table A2:** Hardness of tablets prepared by adding different types of oligosaccharides.

Types of Oligosaccharides	Tablet Hardness (N)
Oligo-xylulose	25.58 ± 1.80 ^bc^
Oligofructose	41.44 ± 1.58 ^a^
Oligoisomaltose	22.59 ± 0.17 ^c^
Fructose	28.04 ± 1.70 ^b^
Oligogalactose	40.15 ± 2.84 ^a^

Note: Different lowercase letters in the same column indicate significant differences between groups (*p* < 0.05).
